# Garlic essential oil‐based nanoemulsion carrier: Release and stability kinetics of volatile components

**DOI:** 10.1002/fsn3.2784

**Published:** 2022-04-20

**Authors:** Hamed Hassanzadeh, Mohammad Alizadeh, Reza Hassanzadeh, Babak Ghanbarzadeh

**Affiliations:** ^1^ Department of Food Science and Technology Faculty of Para‐veterinary Ilam University Ilam Iran; ^2^ Department of Food Science and Technology Faculty of Agriculture Urmia University Urmia Iran; ^3^ 117045 Department of Organic Chemistry Faculty of Science Urmia University Urmia Iran; ^4^ Dina Pharmed Exir Salamat (DPES), Pharmaceutical Co., VS 36‐2b Research and Development Unit Tehran Iran; ^5^ 56947 Department of Food Science and Technology Faculty of Agriculture University of Tabriz Tabriz Iran; ^6^ Department of Food Engineering Faculty of Engineering Near East University Nicosia Turkey

**Keywords:** garlic essential oil, gas chromatography, kinetic, nanoemulsion, release and instability, volatile compounds

## Abstract

An O/W nanoemulsion of garlic essential oil (GEO) at different oil‐to‐emulsion (O/E) ratios (5%, 10%, 15%, and 25%) was formulated to protect the volatile components of GEO. The effects of O/E ratios on the encapsulation efficiency (EE%) of volatile compounds and droplet size of nanoemulsions were studied. The results showed that with increasing in E/O ratio, droplet size increased while EE% decreased so that the droplet size was below 100 nm for all samples and the EE% was almost above 80% for most samples. The effects of various factors such as temperature (5°C–45°C), pH values (3–7), ionic strength (0–500 mM), and O/E ratios (5%–25%) on kinetic of nanoemulsions stability were studied. Reducing pH values and raising the temperature, ionic strength, and O/E ratios intensified the instability process and constant rate of instability in all nanoemulsions. The effects of temperature and O/E ratios on the release kinetics of volatile components were evaluated over time, and kinetic parameters such as release rate constant (k), Q10, and activation energy (Ea) were calculated in which results showed a zero‐degree model to describe the release kinetic behavior of most nanoemulsions. Both temperature and O/E ratios factors as well as their interaction (which had a synergistic effect) had a significant effect on increasing the release rate of volatiles so that the degree of reaction rate was changed from zero to the first order at simultaneous high levels of both factors. FT‐IR spectroscopy was carried out to study interactions among nanoemulsion ingredients. The presence of sulfur‐containing functional groups of garlic oil (thiosulphate, diallyl trisulfide, etc.) in nanoemulsions was confirmed by FT‐IR.

## Graphical abstract

Droplet size increased while EE% decreased so that the droplet size was below 100 nm and the EE% was almost above 80% for most samples. Reducing pH values and raising the temperature, ionic strength, and O/E ratios intensified the instability process and constant rate of instability in all nanoemulsions. The changes in kinetic parameters such as release rate constant (k), Q10, and activation energy showed a zero‐degree model to describe the release kinetic behavior of most nanoemulsions.

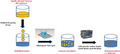

## 1 INTRODUCTION

Kinetic modeling is a very applicable technique for food processing and food quality. The area of kinetics in food systems has received a great deal of attention in past years, primarily due to efforts to optimize or at least maximize the quality of food products during processing and storage. Kinetic modeling enables us to describe chemical, biochemical, and physical reactions and their rates quantitatively (Heldman et al., 2006). Moreover, a good understanding of reaction kinetics can provide a better idea of how to formulate or fortify food products to preserve the existing nutrients or components in a food system. Unfortunately, limited kinetic information is available at present for food systems or ingredients that inhibit the rapid development of new functional food products with improved stability or the optimization of processing conditions (Heldman et al., 2006).

In recent years, nanoemulsions have shown great potential in the encapsulation of lipophilic compounds which can develop the application of essential oil in food formulation (Salvia‐Trujillo et al., 2015). Nanoemulsions are the fine droplet emulsions that have a droplet size below 200 nm and they have shown relatively higher kinetic stability against flocculation, coalescence, and creaming than macroemulsions due to their smaller droplet size (McClements, 2012; Saberi et al., 2013; Salvia‐Trujillo et al., 2015). Fabrication of nanoemulsions for encapsulation and controlled delivery of functional ingredients is one of the functional areas of nanotechnology in the food industry (McClements, 2012; Velikov & Pelan, 2008; Yang et al., 2012).

Researchers reported that the nanoemulsions based on essential oil have antimicrobial activity against positive and negative bacteria, mold, yeast, fungi, viruses, and spores without adding any synthetic or natural antimicrobial additives (Weiss et al., 2009). Regarding the previous results, these nanoemulsions had an antimicrobial potential when they were formed only by water, oil phase, surfactant, and alcohol which is attributed to the electrostatic beyond between cationic charge of nanosize droplets of emulsions and anionic charge of the pathogen cell (Jumaa et al., 2002). Nanosize droplets bind to the outer membrane of the microbial cell by electrostatic attraction and transmit their energy to the outer membrane and consequently disrupt the cells (Donsì et al., 2011; Liang et al., 2012).

Artiga‐Artigas et al. (2018) evaluated the effect of lecithin, Tween 20, and SMP (Sorbitan Monopalmitate) on encapsulation and stabilization of curcumin‐loaded nanoemulsions. They reported that nanoemulsions with 2.0% *w/w* lecithin did not suffer destabilization phenomena during almost 86 days of the experiment, whereas those containing Tween 20 or SMP at the same concentrations were destabilized after 5 days or along 24 hr, respectively. Indeed, Tween 20 concentrations of 0.5% *w/w* showed the fastest release of curcumin.

Md Saari et al. (2020) worked on the release kinetics of encapsulated curcumin based on the cumulative amount of curcumin released from nanoemulsion. The data were analyzed and fitted using five kinetic models such as zero, first, second, Higuchi, and Korsmeyer–Peppas equations. The results showed that both Higuchi and Korsmeyer–Peppas models could fit well into the data satisfactorily. The model achieves the highest correlation coefficient (>0.9) for both free curcumin and encapsulated curcumin.

Doi et al. (2019) studied the impact of garlic oil droplet properties on flavor release from the heated emulsion and explained that higher oil levels increased flavor retention but decreased physical stability and low molecule surfactants had slow flavor release but decreased physical stability. Also, the emulsifier type impacted emulsion stability and flavor retention.

Fabrication of nanoemulsion based on GEO has a great advantage due to covering its unfavorable flavor, especially in a dairy beverage, such as buttermilk, kefir, etc., and maintaining its biological functions. This article aimed to study the release kinetics of volatile components from the nanoemulsion containing essential oil of garlic and to study different factors affecting release phenomena in this type of nanoemulsion.

## 2 MATERIALS AND METHODS

### 2.1 Materials

The essential oil of garlic was purchased from Tehran Zardband Co. Tween 80 and Citric acid were purchased from Sigma‐Aldrich Co. The used materials were analytical grade.

### 2.2 Methods

#### 2.2.1 Nanoemulsion preparation

A combination of low energy and high energy methods was used for the production of nanoemulsions. At first, essential oil of garlic and tween 80 were mixed by magnetic stirrer for 30 min at 500 rpm and then the mixed oil phase (tween 80 and essential oil of garlic mixture) was added slowly to the aqueous phase while mixing by magnetic stirrer for 30 min at 700 rpm (25°C ± 3°C). Then, the produced premixed emulsions were transferred to the water bath ultrasonic with 100 w powers and 40 kHz frequency for 15 min (30°C ± 5°C). The ratio of surfactant‐to‐oil was fixed for all of the nanoemulsions (1:1), while the aqueous phase content was varied (50, 70, 80, and 90%).

#### 2.2.2 Droplet size (Z average) and Polydispersity Index (PDI)

Dynamic light scattering (Malvern Instruments Ltd with precision (range) of measurement 0.6 nm to 6 microns) was used to determine droplet size. All of the nanoemulsions were diluted by distilled water with a ratio of 1:50 to prevent multi‐scattering during droplet size measurement. Experiments were carried out at 25°C and with a 90 angle of refraction.

#### 2.2.3 Encapsulation efficiency

Gas chromatography (Agilent Technologies) equipped with a flame ionization detector (FID) was used to measure encapsulation efficiency. Type of used column was HP5, 50 m × 0.32 mm × 25 μm (19091J‐413) Agilent technologies.INC. Nitrogen was used as carrier gas with a flow rate of 0.7 ml/min. The injection inlet temperature was 120°C and the temperature of the detector was 300°C. Also, the initial temperature of the oven was 40°C and then the temperature increased with a rate of 5°C/min until reached 90°C and was maintained for 2 min at this temperature. Before injection, the samples were heated to 40°C, and then sampling was done from samples headspace (Karagoz et al., 2017). Then, encapsulation efficiency was determined by comparing the total area under the peaks of the nanoemulsions and the not emulsified mixture headspace (Hassanzadeh et al., 2018).

$$\text{EE}\left(\%\right)=\left(1‐\frac{\text{the}\;\text{total}\;\text{area}\;\text{under}\;\text{the}\;\text{peak}\;\text{of}\;\text{the}\;\text{emulsions}\;\text{headspace}}{\text{the}\;\text{total}\;\text{area}\;\text{under}\;\text{the}\;\text{peak}\;\text{of}\;\text{not}\;\text{emulsified}\;\text{mixture headspace}}\right)\times 100.$$

#### 2.2.4 Stability tests

The prepared nanoemulsions were subjected to various conditions to evaluate their stability against gastrointestinal digestion and various storage conditions. The pH value of nanoemulsions was adjusted by HCl (0.1 N) in different states (2, 3, 4, and 6), also NaCl was added to the nanoemulsions in different formulations (0, 250, and 500 mM) and finally, the nanoemulsions were stored at different storage temperatures (5, 25, and 45). The absorbance changes of nanoemulsions were recorded at 500 nm by spectrophotometer (Pharmacia Nova spec II) at 3‐day intervals during storage time to study their stability.

#### 2.2.5 Kinetic analysis

##### Release kinetic of entrapped material

The release of volatile compounds of garlic oil was recorded during storage time by gas chromatography (it was carried out in the way described in the encapsulation efficiency part) (Kiralan et al., 2019). Then, the recorded data were fitted to the zero (Equation 1), first (Equation 2), and second (Equation 3) order reaction, and one of them was selected based on the highest *R* ^2^ and the lowest sum of squared errors (*SSE*).

(1) $$C=C_{0}+kt$$

(2) $$\ln\;C=\ln\;C_{0}+kt$$

(3) $$\frac{1}{C}=\frac{1}{C_{0}}+kt$$

Where *C* is the concentration, t is the time, *C*_0_ is the initial concentration, and *k* is the rate constant (Heldman et al., 2006).

For calculating activation energy (Ea), firstly arranged the calculated reaction rate constant (*K*) at different storage temperatures versus the temperature (in degrees Kelvin), then it was taken a natural logarithm of the reaction rate constant (Ln *K*) and inversed the temperature (1/T).

The sorted data are fitted with the Arrhenius model (Equation 4) and the slope (Ea/R) and the intercept (ln A) are calculated for the next steps.

(4) $$Ln\;K=\frac{‐Ea}{R}\cdot \frac{1}{T}+Ln\;A$$

Also, by substituting a certain constant global amount of gases (8.314472) in the calculated slope (Ea/R), the amount of activation energy (Ea) was determined for each nanoemulsion.

##### Kinetic stability

The absorbance changes of nanoemulsions were recorded during storage time. Then, the recorded data were fitted to the zero (Equation 1), first (Equation 2), and second (Equation 3) order reaction, and one of them was selected based on the highest *R* ^2^ and the lowest sum of squared errors (*SSE*), where c is the absorbance, t is the time, *c*_0_ is the initial absorbance, and *k* is the rate constant (Heldman & Lund, 2007).

#### 2.2.6 FT‐IR analysis

IR spectra were measured with a Fourier transform infrared spectrophotometer (Nexus‐670, Thermo Nicolet, USA) using the potassium bromide (KBr) pellet method. For this purpose, the samples (2 mg) were mixed with KBr (50 mg) to produce the pellet. The analysis was performed within the spectral region of 4000–500 cm^−1^ with 64 scans recorded at a 4 cm^−1^ resolution.

### 2.3 Statistical analysis

All of the experiments were carried out in triplicate at least for mean and standard deviation calculation. Duncan's multiple range tests were used for mean treatments comparison.

## 3 RESULTS AND DISCUSSION

### 3.1 Droplet size and Polydispersity Index (PDI)

The results show that the droplet size is below 100 nm for all nanoemulsions and this may be due to equal content of surfactant and GEO so that the surfactant molecules were able to surround the encapsulated material.

Statistical analysis showed that the droplet size of nanoemulsions was significantly affected by O/E ratios in nanoemulsions formulation (*p* < .05). As shown in Table 1, by increasing the proportion of O/E ratios in nanoemulsion formulations, the droplet size has also increased.

**TABLE 1:** Some of physicochemical characteristics of produced nanoemulsions

Nanoemulsion type	Droplet size (nm)	Polydispersity Index (PDI)	EE (%)
Nanoemulsions containing 5% GEO	2^a^ ± 80	0.29 ± 0.07^a^	1.15^a^ ± 91.63
Nanoemulsions containing 10% GEO	1.5^b^ ± 82	0.25 ± 0.1^a^	1.25^b^ ± 86.33
Nanoemulsions containing 15% GEO	82 ± 1^b^	0.33 ± 0.06^ab^	0.47^c^ ± 84.96
Nanoemulsions containing 25% GEO	92 ± 2.5^c^	0.38 ± 0.09^b^	0.65^d^ ± 77.46

Note

Similar letters show no significant difference in (α = .05).

The increase in droplet size with the increase of GEO concentration could be attributed to the following mechanisms: (1) the increase in the number of GEO droplets can increase the probability of droplet collisions which, in turn, enhances flocculation and coalescence phenomena. (2) Increase in GEO concentration can decrease homogenization efficiency for disruption of oil droplets.

Analysis of variance of data related to polydispersity index shows that except for nanoemulsion containing 25% garlic essential oil, there is no significant difference between the other samples. The polydispersity index for all samples is below 0.4, which is generally in the appropriate range, indicating a uniform particle size.

Maybe it can be noted that there was an equal content of essential oil of garlic and surfactant in nanoemulsion formulation and the surfactant molecules were able to surround the encapsulated material.

Also based on the obtained results, the type of oil used in nanoemulsion formulation had a significant effect on the droplet size at a fixed ratio of the dispersed phase and replacing the essential oil of garlic with sunflower oil increased the droplet size. In recognition of these results, Ziani et al. (2012) encapsulated the functional lipophilic compounds, such as vitamin D, vitamin E, and lemon oil in the surfactants based on colloidal delivery systems and they reported that type of oil and surfactant significantly affected colloidal dispersions characteristics. Also, Saberi et al. (2013) fabricated oil in water nanoemulsions containing vitamin E and optimized droplet size of nanoemulsions by some factors that they also reported that the type of oil used in nanoemulsion formulation affected the size of droplets.

### 3.2 Encapsulation efficiency

**FIGURE 1:**
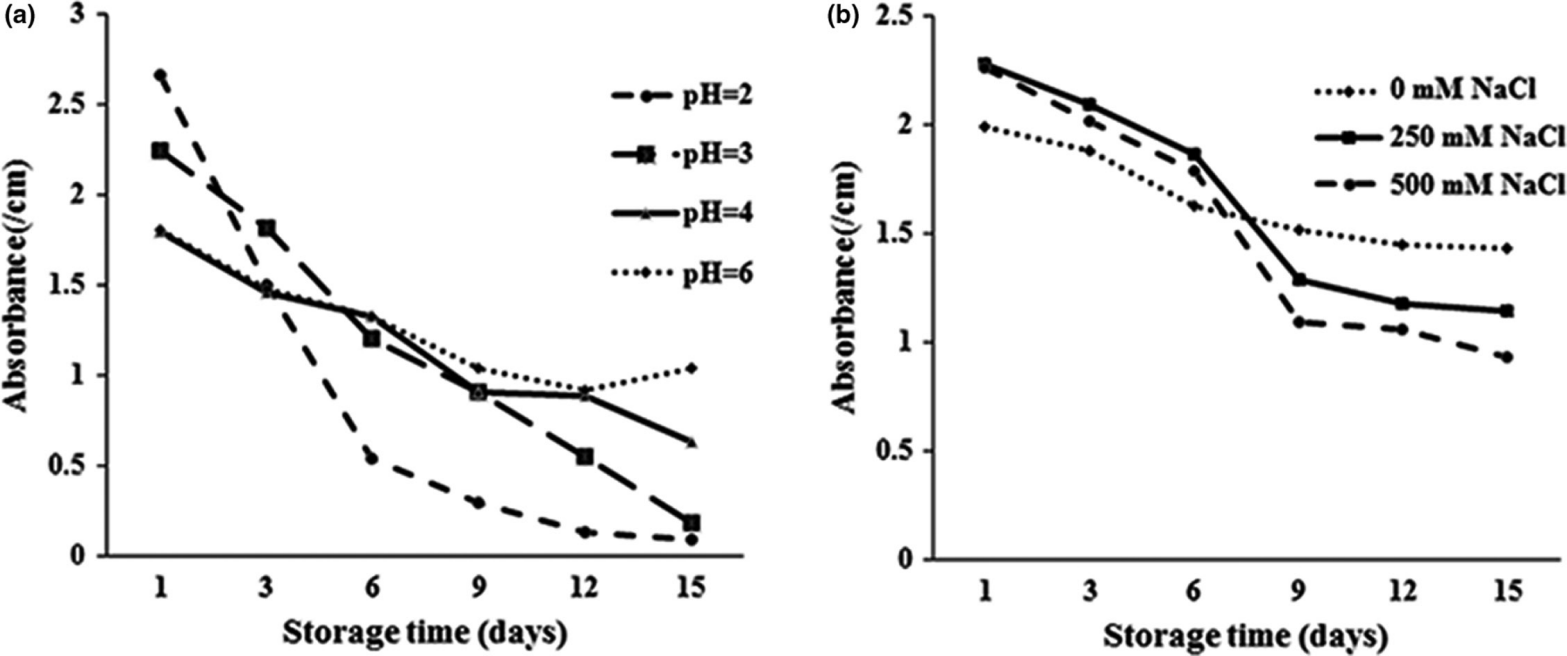
Effect of various pH values (a) and various ionic strength (b) on turbidity changes of nanoemulsions containing GEO during storage time at ambient temperature

As mentioned in the 2.2.4 section, encapsulation efficiency was determined by comparing the total area under the peaks of the nanoemulsions and not emulsified mixture headspace (Figure 1). Based on the obtained results, it was clear that O/E ratios were significantly affected encapsulation efficiency (*p* < .05). The encapsulation efficiency was decreased significantly from 92% to 77% with increasing O/E ratios from 10% to 25% (Table 1) which shows that surfactant molecules were not well able to surround the oil molecules in the higher O/E ratios and especially in the 25%. The present study results are similar to Mazloom and Farhadyar (2014), which reported that encapsulation efficiency was decreased by raising blueberry essential oil content in nanoemulsion formulation. Also, Rachmadi et al. (2015) encapsulated silymarin and curcumin by ultrasonic emulsification method and they reported that the encapsulation efficiency decreased with increasing oil phase percent in nanoemulsion formulation so that increasing the content of silymarin and curcumin from 5 to 35 mg in oil phase (10% w/w) decreased the encapsulation efficiency from 99% to 75% for curcumin and 80% for silymarin.

### 3.3 Effects of factors on the stability of nanoemulsions

#### 3.3.1 Effects of pH on stability of nanoemulsions

**FIGURE 2:**
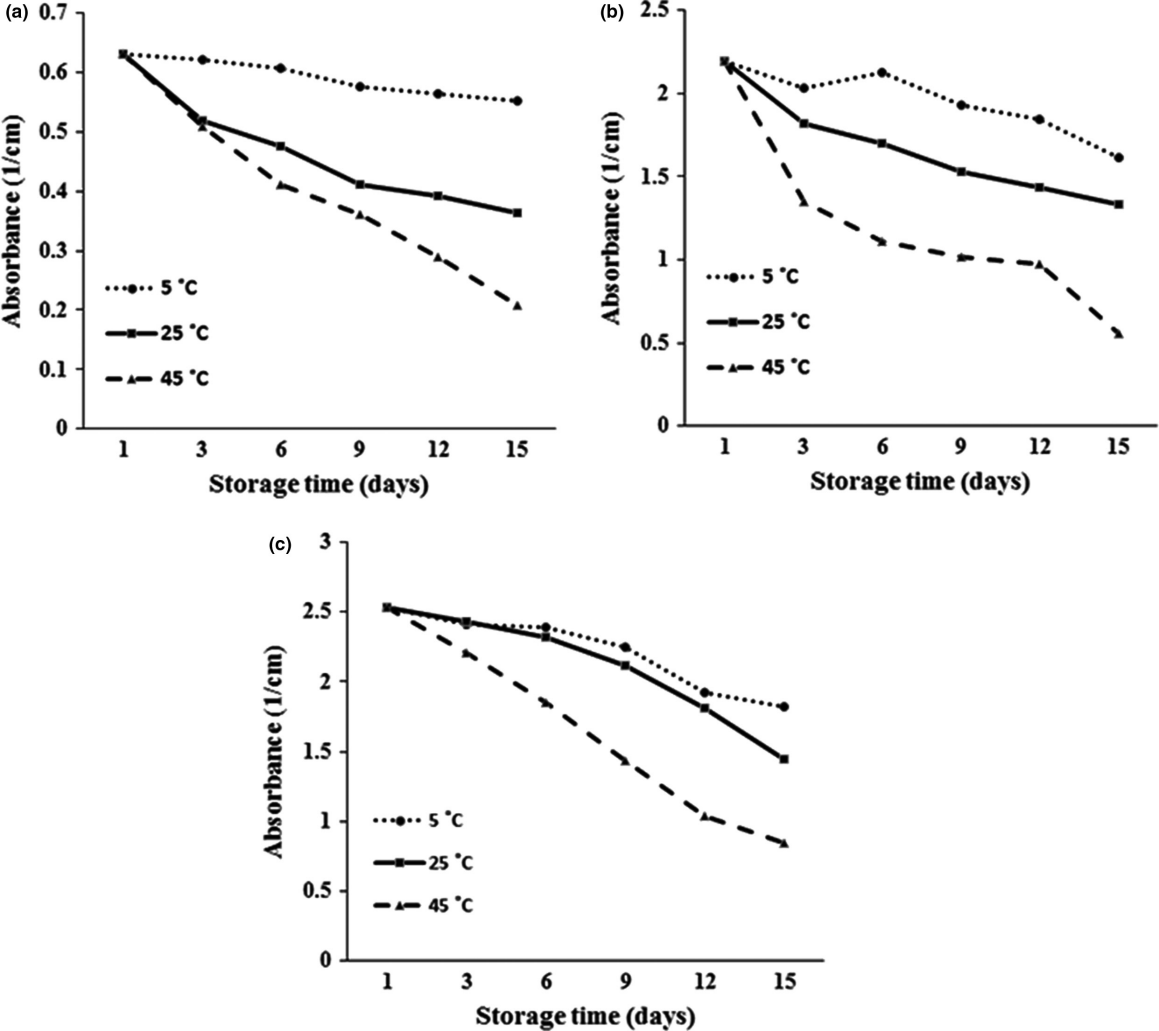
The effect of storage temperature on changes in turbidity of nanoemulsions in various formulations. Nanoemulsion containing 5% GEO (a), Nanoemulsion containing 15% GEO (b), Nanoemulsion containing 25% GEO (c)

The application of nanoemulsions in the formulation of relatively acidic foods such as beverage and dairy products caused the stability of nanoemulsions is considerable in the acidic condition (Qian, Decker, Xiao & McClements, 2012). Also, concerning the condition of human gastrointestinal digestion and particularly the stability of nanoemulsions against the acidic condition of the stomach, studying on effects of various pH values on the stability of nanoemulsions is highly valuable. Results obtained from storage of nanoemulsions containing GEO at various pH values (3–7) and ambient temperature showed that the turbidity of nanoemulsions reduced during storage time and this reducing trend intensified by reducing pH value (Figure 2a).

Reduction of turbidity of nanoemulsions during storage time at ambient temperature may be due to droplet coalescence and/or Ostwald ripening processes and consequently instability of nanoemulsions. It seems that accelerating the instability of nanoemulsions by decreasing pH value is due to reducing the electrostatic repulsion between the oil phase droplets encircled by a surfactant (McClements, 2005). In this situation, the attractive colloidal interactions (van der Waals) overcome the repulsive interactions (steric repulsion generated by hydrophilic head groups of surfactant and electrostatic repulsion) and it leads to droplet coalescence and/or Ostwald ripening processes of the dispersed phase (Rao & McClements, 2013).

Some researches were performed about the effect of pH value on the stability of nanoemulsions which verified the results of the present study. Rao & McClements (2013) examined the influence of various pH values on the stability of nanoemulsions produced by lemon oil and sucrose monopalmitate. They reported that the nanoemulsions with pH values between ranges of 5 and 6 had the most stability while the growth and aggregation of oil phase droplets were observed at higher and lower pH values. Qian, Decker, Xiao, and McClements (2012) studied the physical and chemical stability of nanoemulsions enriched by β‐carotene and declared that the rate of color fading due to degradation of β‐carotene was faster at pH 3 than at pH 4–8. In addition, Lesmes and McClements (2012) evaluated the effect of coating lipid droplets with β‐lactoglobulin–dextran Maillard conjugates on their physicochemical stability and lipase digestibility under simulated gastrointestinal conditions and announced that the most instability of β‐lactoglobulin‐coated emulsions was observed around the isoelectric point (pH 4.8–5.2) of the protein.

#### 3.3.2 Effects of ionic strength on the stability of nanoemulsions

Salts are used in foods, pharmaceutical, and cosmetic formulations, and are present in the human gastrointestinal tract which may affect the functional performance of colloidal systems during their production and after ingestion (Rao & McClements, 2013). In the present study, regarding the presence and influence of salts on the nanoemulsion properties, evaluation of various NaCl concentrations (0–500 mM) on their stability was considered. Results showed that with increasing NaCl concentration and ionic strength, the absorbance of nanoemulsions was reduced with more intensity during storage time, which is indicative of more instability of nanoemulsions (Figure 2b).

The instability mechanism of nanoemulsions with a high concentration of salts in their formulation can correspond to the screening electrostatic repulsion between lipid droplets surrounded by salt ions (McClements, 2005; Qian, Decker, Xiao & McClements, 2012). In lower concentrations of salts, the electrostatic repulsion forces are still so strong to overcome the van der Waals interactions; therefore, precipitation and instability of nanoemulsions were not highly observed. In contrast, in a higher concentration of salts in the formulation of nanoemulsions, particularly in higher than critical concentration point, the electrostatic repulsion forces are not able to overcome the van der Waals interactions; therefore, precipitation and instability of nanoemulsions occurred (Qian, Decker, Xiao & McClements, 2012).

Another researcher also studied the influence of salt added to the nanoemulsions. For instance, Qian, Decker, Xiao, and McClements (2012) examined the effect of various concentrations of NaCl (0–500 mM) on the stability of nanoemulsions containing β‐carotene and reported that the influence of ionic strength on aggregation and precipitation of droplets was considerable. Also they stated that little changes in droplet size were observed in a salt concentration lower than 200 mM, while the aggregation of droplets was also observed in a higher concentration of salt. Rao & McClements (2013) evaluated the influence of NaCl addition (0–200 mM) on the stability of nanoemulsions produced by lemon oil and sucrose monopalmitate and declared that the produced microemulsions containing 0–200 mM were relatively stable, whereas growth and aggregation of droplets were observed in nanoemulsions containing salt higher than 50 mM after 1‐month storage at pH 7. Moreover, Qian, Decker, Xiao, and McClements (2012) reported that changes in color and degradation of β‐carotene were independent of ionic strength (0–500 mM of NaCl), but the lipid droplets surrounded by β‐lactoglobulin were unstable at pH values close to the isoelectric point of the protein (pH 4 and 5), at high ionic strengths (NaCl > 200 mM, pH 7), and at higher storage temperatures (55°C).

#### 3.3.3 Effects of storage temperature on the stability of nanoemulsions

**FIGURE 3:**
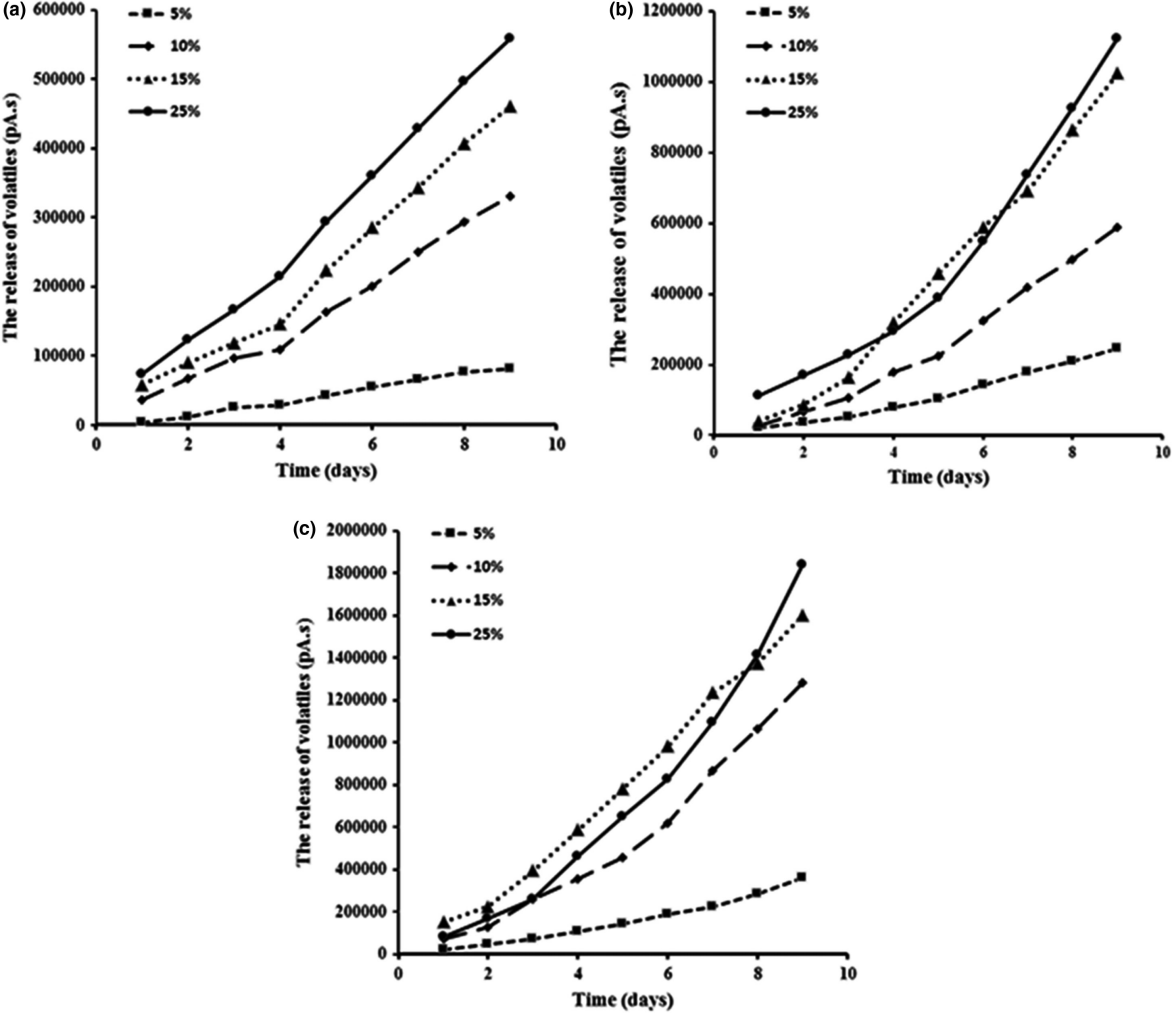
The effect of O/E ratio on volatile component release during storage time at different temperature (24°C=a, 37°C=b, 50°C=c)

In general, colloidal systems may be subjected to various temperatures before their use in commercial applications; for this purpose, studying the effects of various storage temperatures on the stability of nanoemulsions is valuable (Rao & McClements, 2013). In the present study, the influence of various storage temperatures, refrigerator temperature (5°C), ambient temperature (25°C), and elevated temperature to accelerate the effect of temperature on the instability trend of nanoemulsions during storage time (45°C) was evaluated. Results obtained from nanoemulsions produced with different formulations and stored at various temperatures showed that absorbance changes and instability trend of all formulated nanoemulsions with various formulations were faster at higher storage temperatures (Figure 3a–c). Accelerating the instability trend of nanoemulsions can be related to the dehydration of hydrophilic head groups of surfactant at a higher temperature, which may lead to altering the optimum curvature of the surfactant monolayer and the type of colloidal structures formed (Israelachvili, 1992; Rao & McClements, 2013).

Qian, Decker, Xiao, and McClements (2012) evaluated the effects of temperature, pH, ionic strength, and type of emulsifier on physicochemical stability of nanoemulsions enriched by β‐carotene and declared that color fading due to degradation of β‐carotene was accelerated by raising the temperature from 5 to 55°C. Also, Rao & McClements (2012) reported that microemulsions produced by lemon oil and sucrose monopalmitate formed gel when they were stored at 5°C, were relatively stable at 25°C, while growth, aggregation, and precipitation of droplets were observed at 45°C. In addition, they declared that produced nanoemulsions stored at 5°C and 25°C were relatively stable, but nanoemulsions stored at 45°C involved aggregations and precipitation of droplets.

### 3.4 Kinetic stability of nanoemulsions

All of the studied factors (storage temperature, GEO percent, ionic strength, and pH value) significantly affected the changes in turbidity and instability process of nanoemulsions during storage time; therefore, more understanding on kinetic behavior instability of nanoemulsions under influence of studied factors can be considerable. The recorded data of turbidity versus storage time were fitted with zero‐, first‐, and second‐order models and kinetic order, and constant rate of instability process of nanoemulsions, associated with their coefficient of determination, was calculated.

Results obtained from fitting the recorded data of changes in turbidity of nanoemulsions versus storage time with kinetic order models showed that although changes in turbidity of nanoemulsions (instability mechanism of nanoemulsions) followed first and second order in some states, in general, in many cases, the recorded data were fitted with zero order in higher coefficient of determination. It is necessary to note that the negative value of the constant rate of nanoemulsions is indicative of a decline in turbidity of nanoemulsions during storage time, and it can be stated that the instability trend of nanoemulsions followed zero order at ambient temperature. As shown in Table 1, by comparing the obtained constant rates, it can be found that, generally, the constant rate values of changes in turbidity of nanoemulsions were increased by raising the GEO in formulation of nanoemulsions at all temperature storages (5°C, 25°C, and 45°C), which is demonstrative of accelerating the instability trend by heightening the oil phase percent in the formulation of nanoemulsions.

Results from the study on the influence of three factors, storage temperature (5°C, 25°C, and 45°C), pH values (3–7), and ionic strength (0, 250, and 500 mM), on the changes in turbidity of nanoemulsions at fixed O/E ratio (15%) showed that by elevating temperature and ionic strength and reducing pH value, the constant rate of changes in turbidity of nanoemulsions was increased during the storage time (Table 2). By comparing the obtained constant rates and kinetic orders for the three mentioned factors (in studied ranges), it can be understood that the pH values factor (especially at pH 3) more affected the instability trend of nanoemulsions compared to the other two factors (storage temperature and ionic strength).

**TABLE 2:** Effects of studied factors on kinetics parameters and kinetics order

Factors	Kinetics parameters	Model order	Constant rate (day^−1^)	*R* ^2^
Temperature	5°C	Zero	−0.01466	.9513
	25°C	Second	−0.02234	.9866
	45°C	Zero	−0.0245	.9865
pH	3	Zero	−0.1273	.9904
	5	Zero	−0.0557	.9644
	7	Second	−0.01916	.9933
Ionic strength	0 mM	First	−0.03298	.9753
	250 mM	Zero	−0.07878	.9575
	500 mM	Zero	−0.08216	.9846

### 3.5 Kinetic parameters for stability tests

As mentioned before, the storage temperature is another factor that affected the trend of the kinetic change of turbidity of nanoemulsions. As demonstrated in Table 3, the constant rate of all produced formulations was increased by elevating storage temperature which shows the accelerating instability trend of nanoemulsions at elevated temperatures. It is necessary to note that by comparing the obtained constant rate of instability trend of nanoemulsions with various formulations and stored at a different temperature, it could indicate that the storage temperature is the more effective factor than oil phase percent (O/E ratio) on the instability process of nanoemulsions since the storage temperature more increased constant rate of instability process of nanoemulsions than O/E ratio.

**TABLE 3:** Kinetics parameters and Kinetic order of nanoemulsions with different formulations at various temperatures storage

Factors		Kinetic model order	Constant rate (day^−1^)	*R* ^2^	Ea (kJ/mol)
Oil phase (%)	Storage temperatures (°C)				
5	5	Zero	−0.01466	.9723	28.38
	25	First	−0.02234	.9948	
	45	Second	−0.0245	.9919	
15	5	Zero	−0.02961	.9513	16.84
	25	Second	−0.03999	.9866	
	45	Zero	−0.06947	.9865	
25	5	Zero	−0.04313	.9946	15.79
	25	Zero	−0.07030	.9886	
	45	Zero	−0.08558	.9722	

The activation energy was decreased by raising the O/E ratio so that the activation energy was decreased from 28.38 to 15.79 kJ/mol when the O/E ratio increased from 5% to 15%. As the activation energy is defined as the least energy level of molecules which is necessary to progress a chemical reaction and to form a product, the required energy level to progress the instability process of nanoemulsions at high O/E ratios than lower O/E ratios can be understood.

### 3.6 Effect of factors on volatile component release

#### 3.6.1 Effect of O/E ratio on volatile component release

**FIGURE 4:**
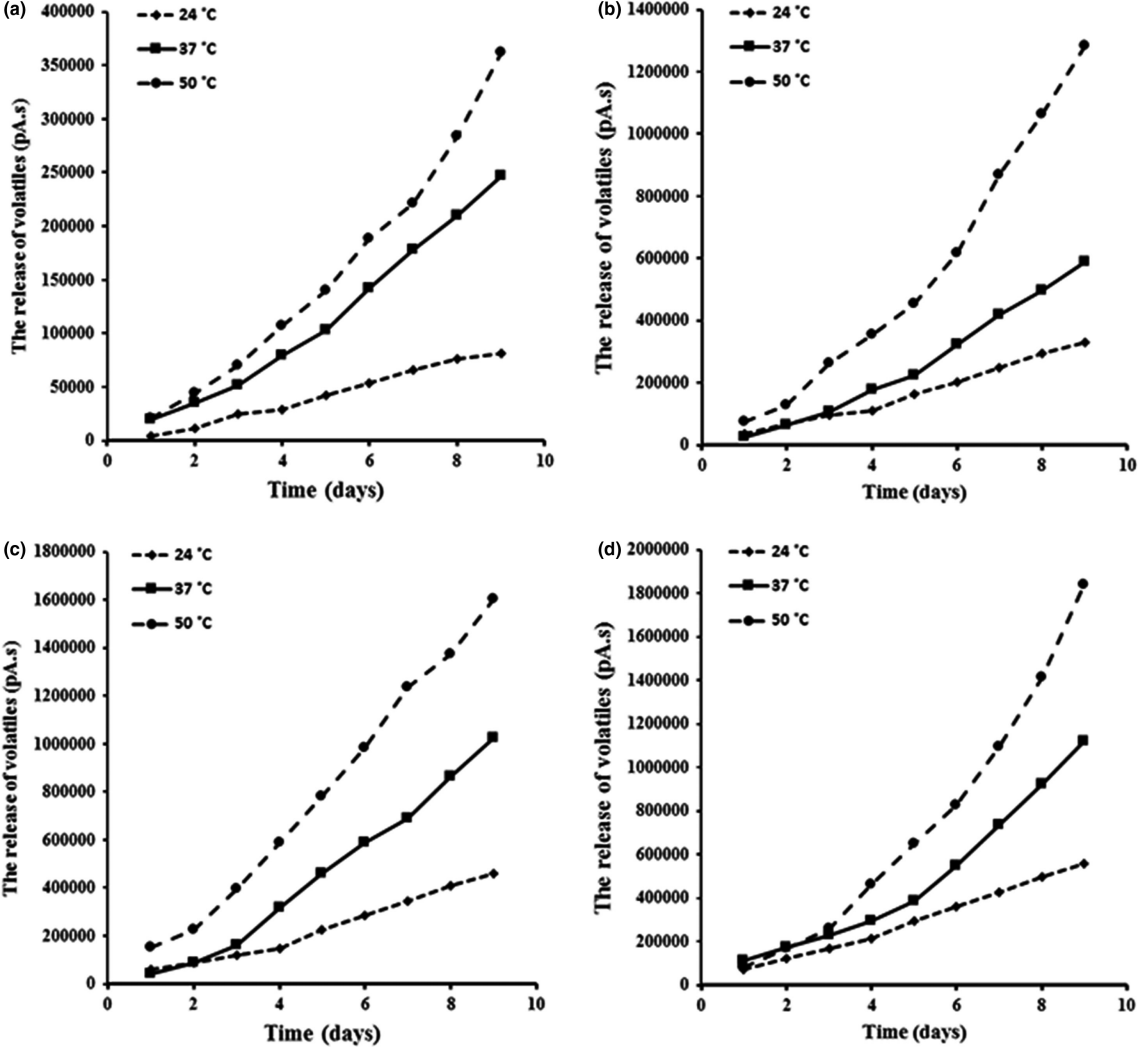
The effect of temperature on volatile component release during storage time for various nanoemulsion (nanoemulsions containing 5% GEO = a, nanoemulsions containing 10% GEO = b, nanoemulsions containing 15% GEO = c, nanoemulsions containing 25% GEO = d)

By analyzing obtained results of volatile components released from inner phase to nanoemulsions headspace, it was clear that O/E ratio (GEO percent) affected volatile component release. As shown in Figure 4, the difference between various nanoemulsion volatile component releases was obvious since first storage time days and this difference was also observed until last storage time days at different temperatures and volatile component releases increased by increasing GEO in nanoemulsion formulation. Increasing volatile component release and their permeation from inner phase to nanoemulsions headspace by raising O/E ratio in nanoemulsion formulation (from 5% to 25%) can be related to encircling GEO molecules (as inner phase) by surfactant (as a connecter bridge between GEO and water molecules) and water molecules (outer phase). Also, increasing GEO concentration causes insufficient encircling inner phase molecules by surfactant and water molecules and consequently increases volatile component release.

Based on the Fick's law, increasing difference concentration (from 5% to 25%) leads to more mass transfer and consequently material immigration facilitation from the primary environment with higher concentration to the secondary environment with lower concentration. It is used first (Fick's law (Equation 5)) when there is a difference in concentration only in one direction for each special ingredient:

(5) $$5m=‐DA\frac{\partial C}{\partial x}$$

In this equation, $\dot{m}$ is the mass flow rate (kg s^−1^) of each ingredient, C is the concentration of each ingredient (kg), D is the diffusion coefficient of each ingredient (m^2^ s^−1^), A is the area (m^2^), and x is the thickness (m) (Cussler, 2009). As it is obvious from this equation, the mass flow rate is increased when the concentration difference of GEO volatile molecules increased between the inner and outer phase of nanoemulsions.

#### 3.6.2 Effect of temperature on volatile component release

Based on the obtained results from measuring volatile component release in oil in water nanoemulsions with various formulations and at different storage temperatures, it is obvious that increasing temperature causes the release and migration acceleration of GEO volatile components to the headspace. The release content of GEO volatile components increased from 500 to 40,000 pA by raising the temperature from ambient temperature (24°C) to 50°C in nanoemulsions containing 5% GEO (Figure 4).

Also, as shown in Figure 4, the volatile component release signal changed from 20,000 pA at first storage days to 140,000 pA (for nanoemulsions with 10% garlic essential oil), to 180,000 pA (for nanoemulsions with 15% GEO), and to 200,000 pA (for nanoemulsions with 25% garlic essential oil) at last storage day by increasing temperature from 24°C to 50°C. By comparing these changes, it can be found that the temperature effect on the release of the volatile components was more significant at a higher O/E ratio, and as it was discussed previously, this issue approved a typical synergetic relation between studied factors (temperature and O/E ratio).

In general, intensifying release and migration of GEO volatile components from inner phase to nanoemulsions headspace by raising temperature can be due to several reasons; the first reason is attributed to reducing nanoemulsions viscosity by increasing temperature that the intermolecular attraction in continuous and O/E ratio was decreased and in turn, it can facilitate release and migration of volatile components from inner phase to nanoemulsions headspace. Also, the second reason is attributed to increasing motional energy so that raising temperature leads to intensify molecule collision energy and entropy of the system and in turn, it can accelerate volatile component release. In other words, it can be expressed that generally, the colloidal systems are the most stable when they have the minimum energy level, and raising systems energy by increasing temperature can lead to heightening entropy and consequently intensifying emulsions instability and it can accelerate the migration of the volatile components from inner phase to outer phase in turn.

Generally, the mentioned issues about the temperature and viscosity role on the volatile component release are justifiable by the Stokes–Einstein equation.

(6) $$D=K\frac{\theta }{\mu }$$

In this equation, D = diffusion coefficient in liquids (m^2^ s^−1^), K= mass transfer coefficient (kg s^−1^), θ = temperature (°C), and µ = viscosity (Pa.s). The diffusion coefficient of droplets in liquids has a direct relation with temperature and an opposite relation with droplets radius. Diffusion coefficient decreases by increasing soluble material concentration due to increasing viscosity in the majority of food solutions. Einstein proposed the Theory of Brownian Motion based on kinetic theory and derived the following expression for self‐diffusivity:

(7) $$D=\frac{\mathrm{RT}}{N_{A}}\cdot \frac{1}{6\pi \eta r_{u}}$$

Where NA is the Avogadro number, T is the temperature, and η is the viscosity of the solution or is the solute radius. Einstein assumed that the solute radius is larger than the solvent radius (Sharma & Yashonath, 2007). According to the above equation, temperature and diffusion coefficient have a direct relation and the relation between viscosity and diffusion coefficient is opposite. Also, it is necessary to clarify that the diffusion coefficient of droplets in liquids has a direct relation with volatile components migration from the inner phase to the outer phase of nanoemulsions.

#### 3.6.3 Release kinetics of volatile components

By fitting the obtained experimental data from gas chromatography to different kinetic order models (zero, first, and second), it was clear that the release kinetics of entrapped volatile components in the inner phase of nanoemulsions containing GEO followed zero order in different O/E ratios and temperatures (Table 4). Although experimental data were fitted with a first‐order model with higher R^2^ in higher O/E ratio (25%) and temperature (37°C and 50°C), generally, the obtained results indicated that release kinetic of volatile components from inner phase to outer phase of nanoemulsions followed zero order, and the release rate of volatile components was independent of the concentration. This may occur in two different situations: (a) when intrinsically the release rate is independent of the concentration of component and (b) when the concentration of the compound is so large that the overall release rate appears to be independent of its concentration (Heldman et al., 2006).

**TABLE 4:** Kinetic parameters of volatile components of GEO from nanoemulsions inner phase in various O/E ratio and temperature

Factors		Kinetic order	*R* ^2^	K (Day^−1^)	E_a_ (kJ/mol)	Q_10_
O/E ratio (%)	Temperature (°C)					
5	24	Zero	.992	10,203 ± 250	43 ± 1.4	‐
	37	Zero	.982	29,073 ± 480	״	2.3 ± 0.2
	50	Zero	.968	41,114 ± 700	״	1.1 ± 0.1
	24	Zero	.985	37,587 ± 750	31 ± 1.1	‐
10	37	Zero	.979	71,897 ± 1050	״	1.5 ± 0.1
	50	Zero	.939	115,040 ± 2100	״	1.3 ± 0.1
	24	Zero	.980	52,527 ± 550	39 ± 1.5	‐
15	37	Zero	.986	125,766 ± 2200	״	1.9 ± 0.2
	50	Zero	.992	188,693 ± 3200	״	1.2 ± 0.1
	24	Zero	.993	62,156 ± 760	37 ± 2.1	‐
25	37	First	.994	126,066 ± 2300	״	1.6 ± 0.1
	50	First	.963	213,213 ± 4900	״	1.4 ± 0.1

Also, by fitting the obtained data of total area under chromatogram picks from nanoemulsion headspace with times data at different temperatures during nanoemulsions storage, it was observed that the release rate constant of volatile components was increased with increasing temperature at a fixed O/E ratio (garlic essential oil) that it can be the result of increasing motional energy and enough energy for molecules collision. It is necessary to note that temperature is not the only factor affecting the release rate constant of volatile components but also other factors such as nanoemulsions viscosity are effective on the release rate constant of volatile components, so temperature indirectly affects the release rate constant again in this condition by decreasing of nanoemulsions viscosity. Low viscosity leads to decreasing intermolecular attraction between continuous phase molecules, surfactant and water molecules, and also between surfactant and GEO molecules, and this can cause more release rate of volatile components.

In general, activation energy is defined as the least energy level of molecules that is necessary to progress a chemical reaction and to form a product. The source of this energy is motional energy of droplets collision and mostly the higher activation energy indicates more dependency of release rate constant (k) to the temperature and positive activation energy means that release rate constant increases by raising the temperature.

Obtained data demonstrated that the activation energy was decreased by increasing the O/E ratio (garlic essential oil) in the formulation of nanoemulsions so that increasing the O/E ratio from 5% to 25% reduced activation energy from 43.05 to 37.87 kJ/mol. This condition means that the lower motional energy and lower energy level for molecules collision are necessary to volatile component release and volatile component release is easier for nanoemulsions with higher O/E ratio at the same condition. This result for activation energy approved the release rate constant and release kinetic order results so that release kinetic order changed from zero order to first order at higher O/E ratios (25%).

Although calculated kinetic parameters are related together and exhibited similar results, each of them has special significance and they offered the effect of temperature and O/E ratio on release phenomena of GEO in distinctive ways. Another kinetic parameter that was calculated for the effect of temperature on release rate constant was Q_10,_ which indicates the effect of temperature on reaction rate. As shown in Table 4, the Q_10_ parameter was decreased from 2.374 to 1.690 by raising the O/E ratio from 5% to 25%.

### 3.7 FT‐IR analysis

**FIGURE 5:**
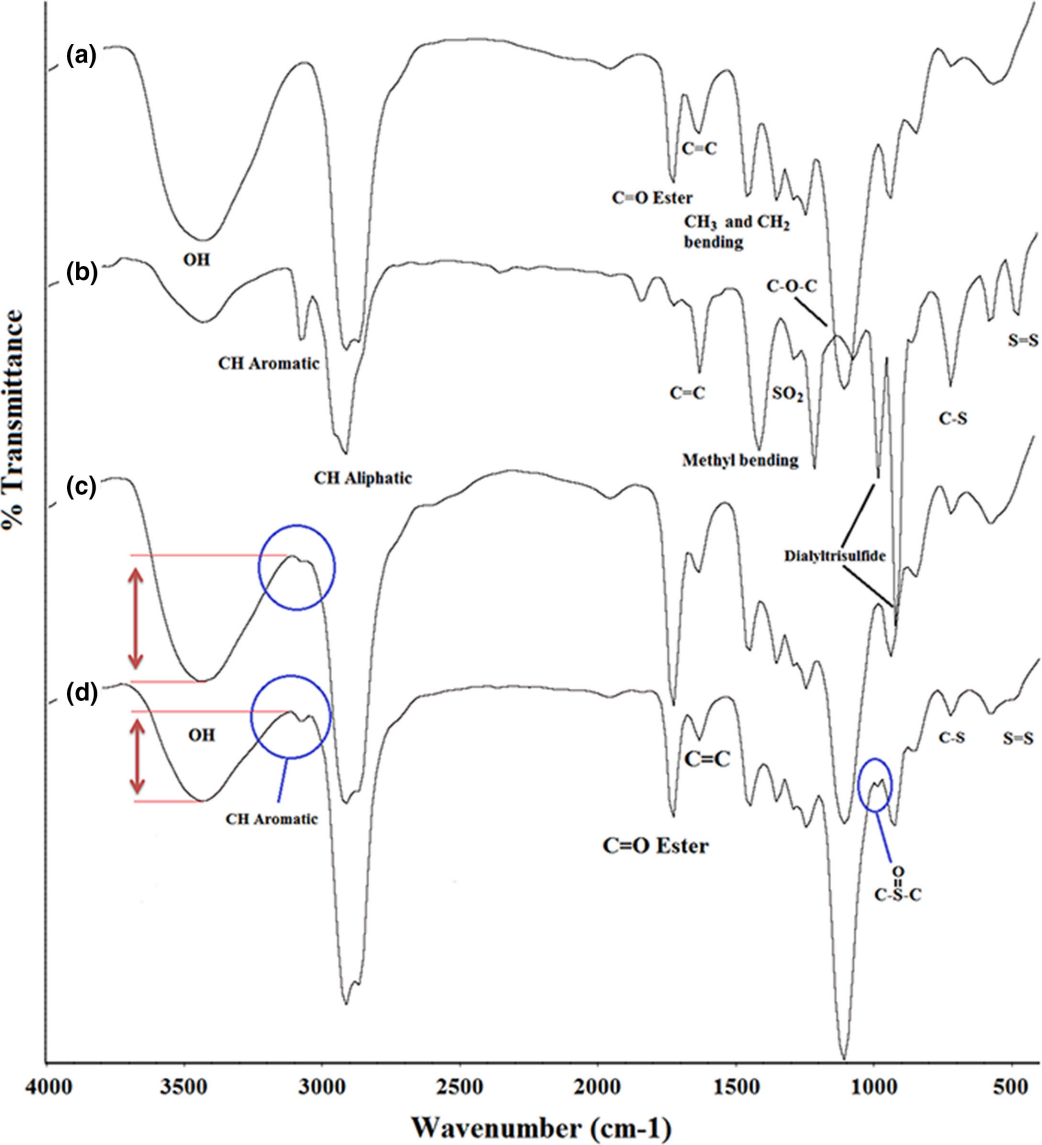
FT‐IR spectroscopy of Tween 80 (a), GEO (b), produced nanoemulsion containing 5% GEO (c) and containing 25% GEO (d)

FT‐IR spectroscopy has been used for the characterization of the samples because of the frequency at which a characteristic group of essential oil compounds is modified by surfactants interactions. Moreover, this technique was used as a tool to investigate the interaction between essential oil, water, and surfactants by measuring the absorption peaks. FT‐IR spectra of the prepared nanoemulsions (5% and 25%) and their constituents (GEO and tween 80 as surfactant) are shown in Figure 5. Figure 5a refers to the tween 80 spectrum which is used as an emulsifier in nanoemulsion formulation. Absorbance peak in the region of 3436 cm^−1^ is attributed to (‐OH) vibrations, and absorbance peak in the regions of 2850–2950 cm^−1^ is related to symmetrical and asymmetrical aliphatic (‐CH) vibrations. The vibrations of C = O ester are shown in the region of 1733 cm^−1^ and the absorbance peak in the region of 1604 cm^−1^ refers to C = C vibrations. The bending vibrations of (‐CH_3)_ and (‐CH_2_) groups are observed in the region of 1459 cm^−1^ and the absorbance peak of 1109 cm^−1^ region is related to the C‐O‐C group (Siddiqui & Ahmad, 2013).

FT‐IR spectrum of GEO is shown in Figure 5b. The absorbance peak of aromatic (‐CH) in the region of 3078 cm^−1^ and the absorbance peak of aliphatic (‐CH) in the regions of 2900–2950 cm^−1^ were observed. The absorbance peak of the 1634 cm^−1^ region is referred to as C = C and the bending vibrations of a methyl group at the 1420 cm^−1^ region are demonstrated. The absorbance peaks of O = S = O vibrations and thiosulphate groups are shown in the regions of 1290 cm^−1^ and 1080–1095 cm^−1^, respectively. The absorbance peaks of stretching vibrations of the C‐(OS)‐C group are exhibited in the region of 1078 cm^−1^ and the absorbance peaks of the 920–990 cm^−1^ region are related to the diallyl trisulfide. Stretching vibrations of the C‐S group are shown in the region of 723 cm^−1^ and the absorbance peaks of 480–495 cm^−1^ are attributed to the S = S group (Siddiqui & Ahmad, 2013; Sun, 2009).

Figure 5c,d are attributed to the nanoemulsions (containing 5% and 25% GEO) spectra and they have a similar spectrum, and the peak intensity in some regions is the only difference between them, which indicates ingredients change in their formulation. As shown in Figure 5c,d, the peak intensity of aromatic (‐CH) vibrations was increased by increasing the GEO percentage in the nanoemulsion formulation. Also, where the GEO percent was lower, the water content would be high, which is verified by (‐OH) group absorbance peak in the region of 3450 cm^−1^. Moreover, the absorbance peaks of S = S and C‐S groups and diallyl disulfide which are related to GEO components have more intensity in 25% nanoemulsions compared to the 5% nanoemulsions.

## 4 CONCLUSION

In general, it can be expressed that volatile components of GEO were covered remarkably by the emulsification method that leads to the longer shelf life of GEO and protects it against oxidation and any other unpleasant changes. The O/E ratio significantly affected droplet size and encapsulation efficiency. Raising the O/E ratio and temperature intensified the volatile component release and migration from inner phase to headspace of nanoemulsions and so decreased the shelf life of nanoemulsions. Also, it can be elicited from results that the lower temperatures are much better to O/W nanoemulsions storage and refrigerator temperature was the best temperature for storage of GEO nanoemulsions. Finally, the results of the present study can offer a new outlook to study more on the release phenomena of nanocarrier and connected kinetic parameters and to more focus on the gas chromatography method for the evaluation of encapsulation efficiency and release content of volatile components as a precise method.

## CONFLICT OF INTEREST

We wish to confirm that there are no known conflicts of interest associated with this publication and there has been no significant financial support for this work that could have influenced its outcome.

## AUTHOR CONTRIBUTION

**Babak Ghanbarzadeh:** Conceptualization (equal); Funding acquisition (equal); Methodology (equal); Supervision (equal). **Hamed Hasanzadh:** Data curation (equal); Formal analysis (equal); Investigation (equal); Writing – original draft (equal). **Mohammad Alizadeh Khaledabad:** Supervision (equal). **Reza Hasanzadeh:** Data curation (equal); Methodology (equal); Software (equal); Validation (equal).

## Data Availability

The data that support the findings of this study are available on request from the corresponding author. The data are not publicly available due to privacy or ethical restrictions.
